# Investigating Structural Property Relationships to Enable Repurposing of Pharmaceuticals as Zinc Ionophores

**DOI:** 10.3390/pharmaceutics13122032

**Published:** 2021-11-29

**Authors:** Oisín Kavanagh, Robert Elmes, Finbarr O’Sullivan, John Farragher, Shane Robinson, Gavin Walker

**Affiliations:** 1 SSPC, The SFI Research Centre for Pharmaceuticals, V94 T9PX Limerick, Ireland; robert.elmes@mu.ie (R.E.); finbar.osullivan@dcu.ie (F.O.); johnpfarragher@gmail.com (J.F.); srobins2@its.jnj.com (S.R.); 2 School of Pharmacy, Newcastle University, Newcastle upon Tyne NE1 7RU, UK; 3 School of Chemical Sciences, Bernal Institute, University of Limerick, V94 T9PX Limerick, Ireland; 4 Department of Chemistry, Maynooth University, National University of Ireland, W23 F2H6 Maynooth, Ireland; 5 National Institute for Cellular Biotechnology, Dublin City University, D09 NR58 Dublin, Ireland; 6 Janssen Pharmaceutical Sciences, T45 P663 Cork, Ireland

**Keywords:** zinc, ionophore, drug repurposing, CSD analysis, ionophorism

## Abstract

The importance of zinc in biology has gained greater recognition in recent years due to its essential contributions to the function of many endogenous enzymes. Disruption of zinc homeostasis may be useful in treating pathological conditions, such as Alzheimer’s, and for antiviral purposes. Despite the growth of knowledge and increased interest in zinc, little is known about the structure and function of zinc ionophores. In this study we analyse the Cambridge Structural Database and solution complexation studies found in the literature to identify key functional groups which may confer zinc ionophorism. Pharmaceuticals, nutraceuticals and amino acids with these functionalities were selected to enable us to explore the translatability of ionophoric activity from in vitro assays to cellular systems. We find that although certain species may complex to zinc in the solid and solution states, and may carry ions across simple membrane systems, this does not necessarily translate into ionophoric activity. We propose that the CSD can help refine key functionalities but that ionophoric activity must be confirmed in cellular systems.

## 1. Introduction

The complexation of pharmaceuticals with metals is one strategy to improve the therapeutic and physicochemical properties of selected pharmaceuticals. Many products available on the market are produced using this method: Pepto-bismol^®^ (salicylic acid and bismuth), Polaprezinc^®^ (carnosine and zinc) and Zyneryt^®^ (erythromycin and zinc acetate). Despite this, it is unclear whether the metals involved in complexation have favorable properties in their own right or the therapeutic activity is conferred through synergy. Reformulation of drugs as metal ion complexes could lead to new, cost-effective therapeutic opportunities (as the safety characteristics for repurposed pharmaceuticals are well established relative to novel compounds)—a consideration that is gaining increased importance as the cost of drug development is expected to exceed $3 bn if it continues to follow recent trends [1].

Zinc is the only metal that is found in all six classes of enzymes established by the International Union of Biochemistry [2]. It is no surprise to find that most endogenous zinc is bound and not freely available [2]—in fact, free zinc is highly toxic, and in the brain extracellular concentrations are as low as 10 nM [3]. One study reported that although the antiviral properties of zinc have been identified in vitro, the concentrations employed are often orders of magnitude larger than those encountered in vivo and that this would be expected to result in cellular toxicity [4]. Zinc toxicity is thought to contribute to the pathophysiology of conditions such as Alzheimer’s, and the success of strategies which attempt to restore homeostasis by chelating zinc (thereby reducing intracellular concentrations) has led to an emerging role for zinc-based therapeutics [3,5]. Frederickson et al. highlight that “*weakly or moderately bound zinc complexes may be useful in altering zinc homeostasis in the cell*” [3,5].

When faced with a pathogenic challenge, human cells fight for supplies of trace elements, such as zinc and iron, which are essential for cellular growth and development [6]. In bacteria, the transport of ions across the lipophilic bacterial membrane is a particular challenge and they have evolved to synthesise and release a variety of metabolites which—when isolated—reveal a remarkable specificity for certain ions [7]. Researchers soon manipulated this mechanism to deliver antimicrobials, and work in this area has resulted in the first FDA approved ionophore drug cefiderocol in 2019 (approved by the EU in April 2020). Indeed, it is possible that many antibiotics possess some ionophoric activity.

It is thought that ionophores function by neutralising the charge of ions through the formation of complexes. These neutral complexes, in addition to the lipophilicity of the ionophore, can enable the transport of ions across a cell membrane corresponding to a concentration and electrochemical gradient [8]. Therefore, a compound must possess certain properties to enable ionophorism: (1) an ability to complex to the ion of interest, (2) a certain degree of lipophilicity to transport the ion across membranes and, for the purposes of therapeutics, (3) these effects should be replicated in complex living systems, such as cells.

Synthetic ionophores have their origins in the work of Pedersen [9] and since his receipt of the Nobel prize, the field of synthetic complexing agents has rapidly expanded; modern species find utility for their anticancer properties [10] and trace metal detection [11]. Despite this expansion of knowledge and the growing interest in zinc [2,3,12], little is known about the structure and function of potential ionophoric compounds [7]. This is apparent as much of the zinc ionophore literature explores the activity of well-known zinc ionophores (e.g., clioquinol, quercetin, pyrithione, PBT2 and 8-hydroxyquinoline) [13,14,15,16,17]. Inspiration can be taken from other fields where Puerta et al. systematically developed a range of bioinspired zinc chelators as matrix metalloprotease inhibitors [18] and Vaden et al. produced a zinc selective anticancer ionophore inspired by naamidine A [19]. Once found, zinc ionophores can be tested with a variety of established in vitro models. Zinc ion transport can be screened with liposomal models employing membrane permeable fluorescent probes which are sensitive for zinc (FluoZin-3) and in vitro toxicity and transport studies can determine if these effects translate to cells within a non-toxic range using a relevant cell model, such as A549 epithelial lung cells.

Screening of ionophores to transport ions across membranes will be assessed in model membranes using liposomes prepared by extrusion of 1-palmitoyl-2-oleoyl-sn-glycero-3-phosphocholine. Liposomal systems are classic membrane mimetics; these systems are impermeable to ions [20] but can be loaded with ion sensitive fluorescent probes, such as FluoZin-3 (a zinc sensitive probe), which can detect zinc ions as they are transported across the cell membrane [21]. Pressman noted that ionophores will obey the Nernst equation [8] and, as such, the liposomal model may underestimate ionophoric effects, particularly in the cases of symport or antiport ionophoric mechanisms, as the ions needed to maintain equilibrium may not be present or indeed may be spent (i.e., transported to the other side of the membrane). For example, clioquinol can also transport protons, which can help sustain the electrochemical environment within the cell, enabling further transport [8].

As cellular systems are often more complex, successful candidates will be screened again using a range of cell lines representing therapeutically relevant tissue types to validate the simpler model. A549 lung epithelial cells will be employed as we envision that one use for zinc ionophores is topical delivery to the lung for antiviral purposes. This is particularly pertinent, as sub-micromolar concentrations of free zinc are known to have significant toxicity [4].

Despite growing interest in zinc ionophores, as evidenced by recent clinical trials (NCT04371406, NCT04334512, NCT04335084 and NCT04377646), a design approach for this ion for pharmaceuticals is scarce in the literature. Structural property relationships can be established with the Cambridge Structural Database (CSD), allowing us to probe the solid state. The CSD has been used previously to analyse a variety of zinc complexes [22,23,24,25], and from this (and our own analysis) we will select a number of compounds that represent key functional groups that enable complexation with zinc. We will then probe their ionophoric activity using in vitro models commonly used in the literature. These hits will then be investigated in a therapeutically relevant cell model (A549 lung epithelial cells) to understand their translatability. In the field of ionophorism, this step is often overlooked in favour of mechanistic studies dedicated to the discovery of new probes and sensors.

## 2. Materials and Methods

Hydroxychloroquine sulfate (HCQ) was received as a gift from Associate Professor Lidia Tajiber (Trinity College Dublin), Clioquinol was purchased from Tokyo Chemical Industries (Oxford, UK) and cholesterol and zinc chloride were purchased from FluoroChem (Glossop, UK). The extrusion kit and materials required to create FluoZin-3 loaded liposomes, such as membrane supports, 0.2 µm polycarbonate membrane filters, FluoZin-3 (membrane impermeable) and 1-palmitoyl-2-oleoyl-glycero-3-phosphocholine (POPC), were purchased from Avanti Polar lipids (Birmingham, AL, USA). All other solvents, Active Pharmaceutical Ingredients (APIs) and materials were purchased from Sigma-Aldrich (Darmstadt, Germany) at the highest grade available.

### 2.1. Cambridge Structural Database

The Cambridge Structural Database was searched using ConQuest (v5.40, August 2021) and the retrieved entries were subsequently analysed with Mercury. Structures containing zinc were selected based on the following criteria: contact inter/intra-molecular bonding (separated by 1–3 bonds 0.5–4 Å), R-factor < 0.05, non-disordered, no errors, single crystal structures and organometallic.

### 2.2. Synthesis of FluoZin-3 Loaded Liposomes

Liposomes were synthesised according to a previously reported procedure [21,26], where 250 mg (3.289 × 10^−4^ moles) 1-palmitoyl-2-oleoyl-glycero-3-phosphocholine (POPC) and 1.4125 mg (3.65 × 10^−6^ moles) cholesterol were dissolved in 8 mL chloroform and 4 mL of this solution was aliquoted into a 25 mL Round Bottom Flask (RBF). The solvent was then removed by rotary evaporation to obtain a thin film. Then, 0.5 mL DMSO containing 590.3 µM of FluoZin-3 was mixed with 3.5 mL PBS buffer (0.01 M PBS, at pH 7.4) and added to the RBF containing the lipid film and subsequently vortexed for 15 min. The solution was subjected to 15 freeze-thaw cycles and subsequently passed through a Sephadex G100 column (previously swelled for 24 h with PBS) to remove any unencapsulated FluoZin-3 from the solution.

This liposomal solution was then extruded 25 times through a 0.2 µm polycarbonate membrane using an Avanti extrusion kit and then stored in a screwcap glass vial covered with tinfoil and kept in the fridge (2–8 °C) until use.

### 2.3. Liposomal Penetration Assay

Stock solutions of liposomes, Active Pharmaceutical Ingredients (APIs) and zinc chloride were prepared and allotted and made up to 2.5 mL in PBS buffer (0.01 M PBS, at pH 7.4) to create a final solution containing 52 µM lipid to 0.1 mM of API (1 mM was used in the case of HCQ, aciclovir, 8-hydroxyquinoline, zinc gluconate, all imidazoles and tryptophan) and 0.1 mM zinc. Fluorescence was determined using a quartz cuvette in a Varian Eclipse fluorospectrometer exciting and emitting at 494 nm and 519 nm; slits were set to 5 nm (determined empirically). During the experiment the fluorescence measurement was started for some minutes to obtain a baseline. Then zinc was added, followed by API and the fluorescence intensity was recorded.

### 2.4. Cell Culture Conditions

Tissue culture was performed with aseptic techniques [27,28]. Exponentially growing type II lung epithelial cells (A549 ATCC^®^ CCL-185) were cultivated at 37 °C under 5% CO_2_, in 1:1 DMEM:F-12 medium (Sigma-Aldrich, Darmstadt, Germany, D8437) supplemented with 10% foetal bovine serum (FBS, Gibco, Darmstadt, Germany, 10270-106) according to ATCC^®^ recommendations. Cells were passaged approximately every 3–4 days, to prevent cells from reaching confluence. Subculture was carried out by first washing the cell monolayers with phosphate buffered saline (PBS), followed by incubation with TrypLE Trypsin-EDTA (0.05%, Gibco, 25300-062) at 37 °C as above. Cell number and viability were determined by staining with trypan blue and counting with a haemocytometer.

### 2.5. Cell Toxicity Assay

A549 cells were plated at a density of 1 × 10^2^ cells per well (for 7 days) and at 1.4 × 10^2^ cells per well (3 days). After allowing 24 h for attachment, agents were aliquoted to the wells to reach final concentrations of 10, 5, 2.5, 1.25 and 0.625 µM (1, 0.5, 0.25, 0.125, 0.625 µM for pyrithione) and incubated for 3 and 7 days. Cell viability was assessed by incubating with Alamar Blue HS (Invitrogen A50101). After incubation, 20 µL of Alamar Blue (final concentration 10% *v*/*v*) was added to each well and the plates were further incubated for 2 h. Fluorescence intensity was recorded with excitation at 530 nm and emission at 590 nm. These conditions were determined empirically, and this procedure was repeated to generate three biological repeats with a minimum of 6 technical repeats in each set.

### 2.6. Fluorescence Imaging

A549 cells were plated in a 24-well plate at a density of 3 × 10^4^ per well. After allowing 24 h for attachment, the APIs were added to the wells to give a final concentration of 2.5 or 300 µM (0.125 and 15 µM for pyrithione); some wells were spiked with 10 µM zinc and further incubated for 24 h. The wells were then washed with PBS and stained with FluoZin-3 AM (Invitrogen F24195) and Pluronic-24 (final concentration of FluoZin-3 AM was 1 µM). Nuclei were counterstained with NucBlue Live (Invitrogen R37605) and further incubated for 1 h. The cells there washed again with PBS and 250 µL serum free media (DMEM:F12) was added to each well before imaging. Cells were imaged using a Nikon TiE equipped with Photometrics Coolsnap HQ2 camera (Tucson, AZ, USA) and controlled by Metamorph (San Jose, CA, USA). These methods have been described previously [29].

## 3. Results

### 3.1. Identification of Zinc Chelators with CSD Analysis

**Figure 1:**
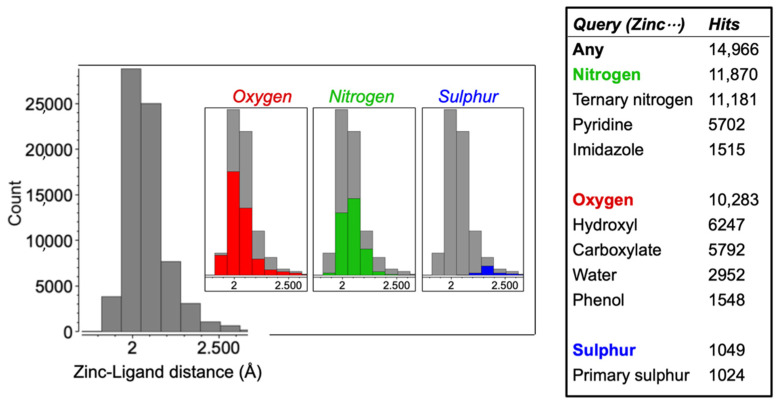
The distribution of zinc–ligand distances identified from the CSD, grouped by the elements bound to zinc within the ligand, and a summary of results from the Cambridge Structural Database detailing the number of hits of zinc solid-state complexes, stratified by chemical functionality.

The Cambridge Structural Database was mined to identify the common functional groups in the solid state. Preliminary searches found that at least one nitrogen, oxygen and sulphur containing functionality is involved in the vast majority of complexes with zinc (Figure 1). Of the total number of zinc structures (n = 14,966), 11,870 contained zinc bound to nitrogen, where almost all of the nitrogen–zinc compounds are bound to ternary nitrogen groups (11,181/11,870; 94%). Further analysis revealed a large diversity of nitrogen containing binding groups with the largest portions containing pyridines (n = 5702/11,870; 48%) and imidazoles (n = 1515 /11,870; 13%). The CSD contained 10,283 structures with zinc bound to oxygen; 2952 of these structures (29%) contained water and 5792 contained carboxylate functionalities (56%). Hydroxyl groups account for the majority of zinc binding to oxygen (n = 6247/10,283; 61%), and phenol functionalities make up a significant fraction of this group (n = 1548). Of the sulphur compounds bound with zinc (n = 1049), primary sulphur groups accounted for the vast majority of structures identified (n = 1024/1049; 98%). This data has been summarised in Figure 1.

**Figure 2:**
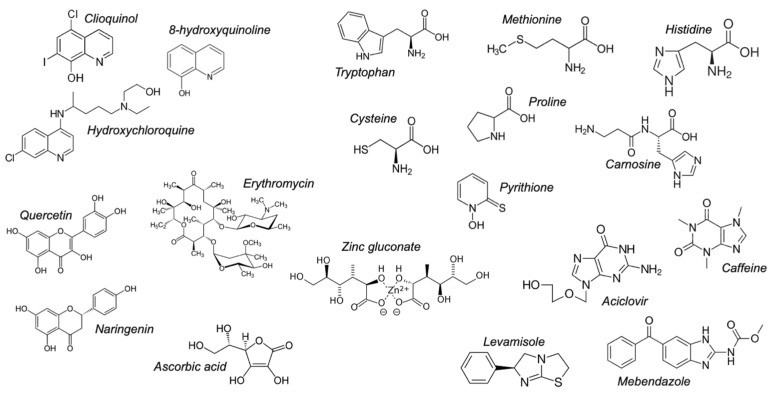
Pharmaceuticals, nutraceuticals and amino acids used in this study.

This analysis helped identify a range of compounds containing nitrogen, oxygen or sulphur functionalities (Figure 2). In the literature, many of these compounds have hypothesised synergy with zinc, such as levamisole [30], quercetin [31], hydroxychloroquine [17] and clioquinol [32], or are currently formulated with zinc for therapeutic purposes, such as erythromycin (Zyneryt^®^), carnosine (Polaprezinc^®^), pyrithione (Head & Shoulders^®^ shampoo) and ascorbic acid (e.g., Beeline^®^ Vitamin C + Zinc).

### 3.2. Liposomal Penetration Behaviour of Selected Compounds

#### 3.2.1. Quinolines

**Figure 3:**
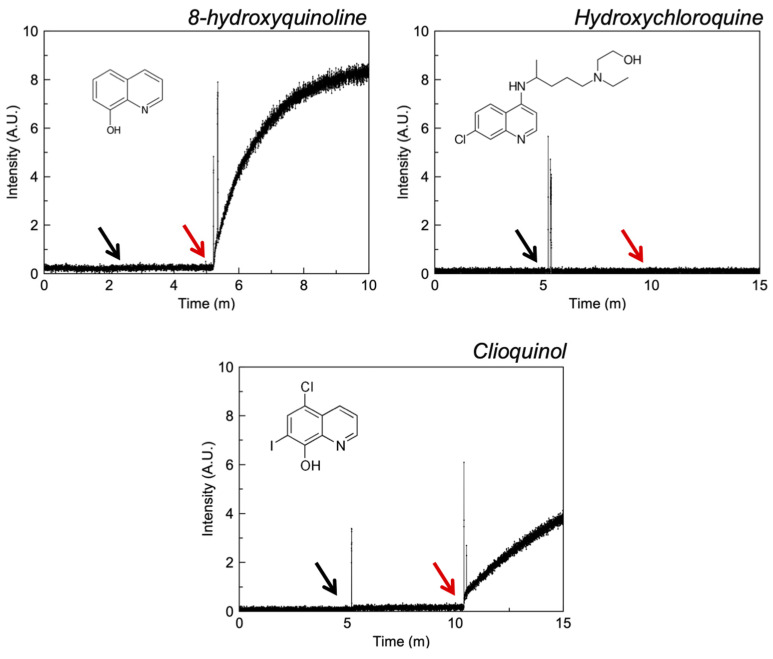
Fluorescence intensity of liposomal FluoZin-3 in PBS (0.1 M, pH = 7.4) before and after the addition of zinc and selected quinolines. The black arrow indicates the addition of zinc and the red arrow the addition of API.

A liposomal ionophorism model with the zinc sensor FluoZin-3 provides direct evidence of these chemical species to transport zinc. Figure 3 reveals that CQL and 8-HQ exhibit ionophoric activity at 0.1 and 1 mM respectively and HCQ possesses no activity even in the presence of excess zinc (1 mM). The potency of CQL with respect to 8-HQ may be explained by the greater partition coefficient of CQL or indeed by the presence of the chlorine electron withdrawing groups on CQL.

#### 3.2.2. Flavonoids 

**Figure 4:**
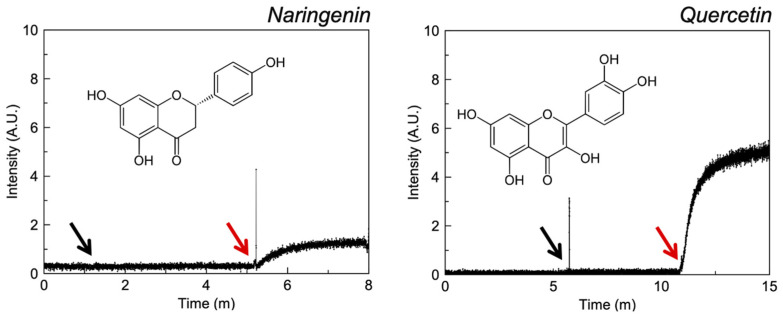
Fluorescence intensity of liposomal FluoZin-3 in PBS (0.1 M, pH = 7.4) before and after the addition of zinc and selected flavonoids. The black arrow indicates the addition of zinc and the red arrow the addition of API.

Figure 4 suggests that quercetin is a more potent zinc ionophore than naringenin, requiring concentrations of 0.1 and 1 mM respectively to produce the responses shown. This has been confirmed previously in liposomal assays prepared via a different methodology where quercetin again demonstrated significantly greater ionophoric activity than naringenin [21,31]. The lack of a hydroxyl group on naringenin may confer weaker affinity for zinc and may explain this difference in effect.

#### 3.2.3. Polyols

**Figure 5:**
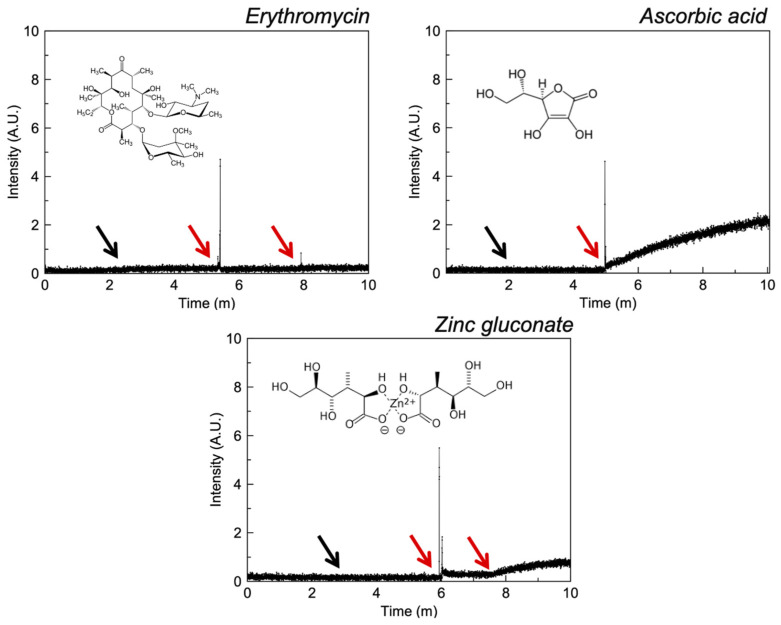
Fluorescence intensity of liposomal FluoZin-3 in PBS (0.1 M, pH = 7.4) before and after the addition of zinc and selected polyols. The black arrow indicates the addition of zinc and the red arrow the addition of API.

Liposomal assays (Figure 5) reveal that ascorbic acid demonstrates ionophoric effects at 0.1 mM concentrations whilst zinc gluconate requires 1 mM concentrations to induce weak ionophoric activity. Erythromycin does not exhibit ionophoric activity up to 1 mM of zinc and erythromycin. Erythromycin is applied as a complex with zinc acetate in a mixture of diisopropyl sebacate and ethanol directly onto the site of action. As such, the complex may be expected to exist in this solvent system when applied to the skin and this environment may enable ionophorism. These conditions cannot be replicated in liposomal models.

#### 3.2.4. Amino Acids

**Figure 6:**
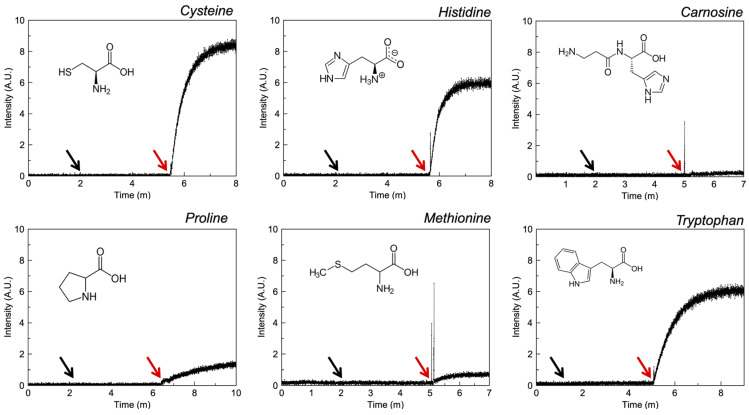
Fluorescence intensity of liposomal FluoZin-3 in PBS (0.1 M, pH = 7.4) before and after the addition of zinc and selected amino acids. The black arrow indicates the addition of zinc and the red arrow the addition of API.

With the exception of carnosine, liposomal studies (Figure 6) suggest that all amino acids are capable of demonstrating at least some ionophoric activity. Cysteine and histidine required 0.1 mM, but proline, methionine and tryptophan required 1 mM to elicit a response. For sulphur containing amino acids, these results are unsurprising as cysteine is the only structure with a primary sulphur (previous CSD analysis illustrated that this accounted for 98% of complexes in the solid state), whereas carnosine proves an exception to the idea that imidazole groups should confer ionophoric activity. A degree of aromaticity, which may increase the basic characteristics of the nitrogen on the five-membered ring appears to be important for zinc transport, and is illustrated by contrasting ionophorism between proline, tryptophan and histidine.

#### 3.2.5. Imidazoles

**Figure 7:**
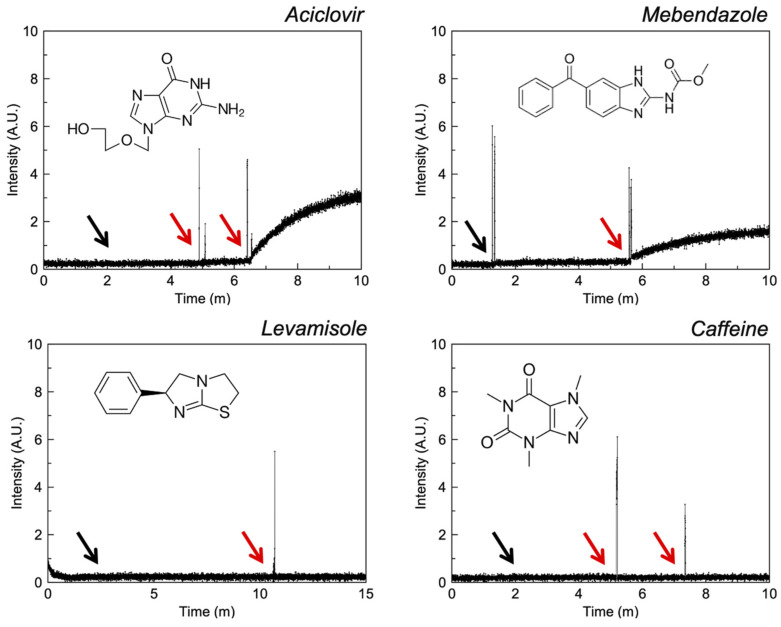
Fluorescence intensity of liposomal FluoZin-3 in PBS (0.1 M, pH = 7.4) before and after the addition of zinc and selected imidazole compounds. The black arrow indicates the addition of zinc and the red arrow the addition of API.

The ionophoric activity of selected imidazoles is demonstrated in Figure 7. Aciclovir and mebendazole illustrate weak ionophoric activity in the presence of 1 mM API and there is an absence of activity for levamisole and caffeine. Perhaps the proximity of an additional H-bond donor to the chelating imidazole nitrogen in mebendazole and aciclovir confers increased complexation strength and provides enough stability to carry ions across the lipophilic membrane.

#### 3.2.6. Miscellaneous

**Figure 8:**
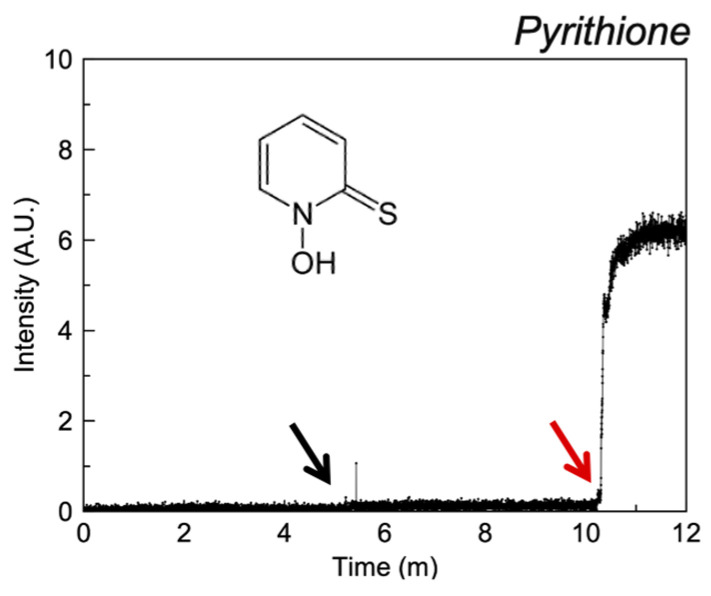
Fluorescence intensity of liposomal FluoZin-3 in PBS (0.1 M, pH = 7.4) before and after the addition of zinc and pyrithione.

Pyrithione elicits a response expected of a strong ionophore in liposomal studies (Figure 8) at 0.1 mM; these effects have been described previously [33,34,35,36].

Table 1 provides a concise summary of the results obtained from experimental data and literature values, along with important physicochemical properties. This highlights that clioquinol, quercetin, cysteine, histidine and pyrithione are the most potent zinc ionophores in liposomal assays, but only pyrithione, clioquinol and hydroxychloroquine affected intracellular zinc concentrations in cellulo.

### 3.3. Cellular Ionophorism with Lead Compounds

**Figure 9:**
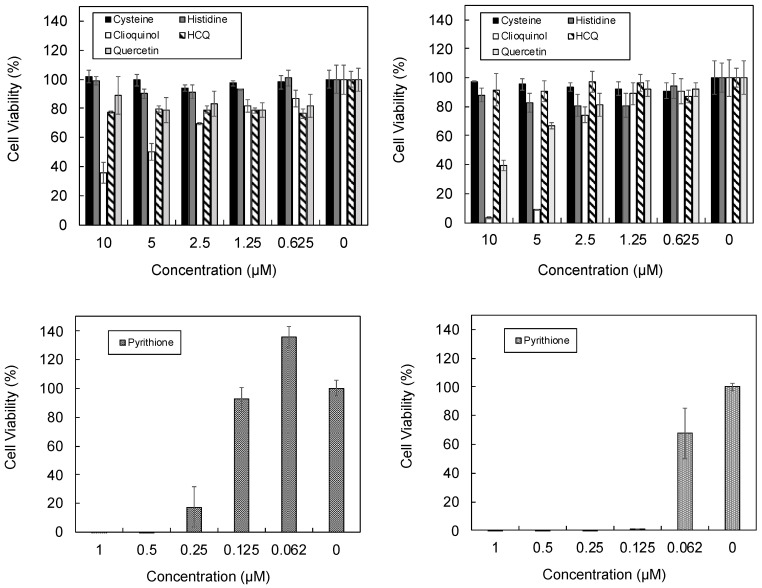
Cell viability assay performed using Almar blue after A549 cells were incubated with drug for 3 (left) and 7 (right) days.

*In cellulo* assays were performed using a lung epithelial line (A549) with the lead compounds identified from the earlier analysis. Cell toxicity assays (Figure 9) were performed to determine the maximum concentrations of drugs that could be tolerated by A549 cells without a large impact on cell viability. It was found that at concentrations higher than 2.5 µM for clioquinol (and 0.125 µM for pyrithione), cell viability was significantly reduced at both incubation times. Thus, 0.125 µM and 2.5 µM was selected as the concentrations of pyrithione and the other compounds to be used in the zinc uptake assays.

**Figure 10:**
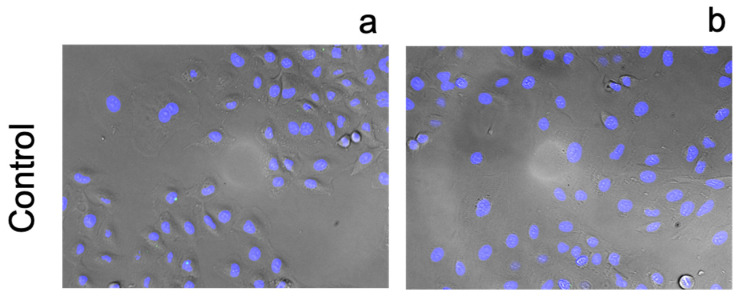
Fluorescence images of A549 cells incubated stained with FluoZin-3 AM (green) and counterstained using NucBlue Live for nuclei (blue) after 24 h incubation with (**a**) basal media and (**b**) with an additional 10 µM zinc chloride added to the media. Magnification ×10.

**Figure 11:**
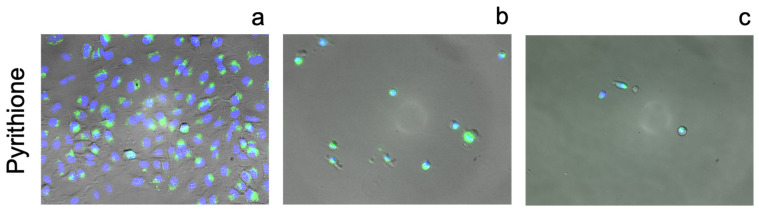
Fluorescence images of A549 cells incubated stained with FluoZin-3 AM (green) and counterstained using NucBlue Live for nuclei (blue) after 24 h incubation with: (**a**) 0.125 µM pyrithione and basal zinc, (**b**) 0.125 µM pyrithione with an additional 10 µM zinc chloride added to the media and (**c**) 15 µM pyrithione with an additional 10 µM zinc chloride added to the media. Magnification ×10.

**Figure 12:**
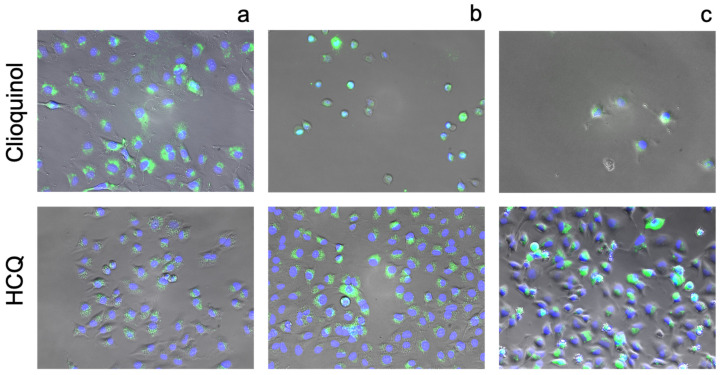
Fluorescence images of A549 cells incubated stained with FluoZin-3 AM (green) and counterstained using NucBlue Live for nuclei (blue) after 24 h incubation with: (**a**) 2.5 µM clioquinol or HCQ and basal zinc, (**b**) 2.5 µM clioquinol or HCQ with an additional 10 µM zinc chloride added to the media and (**c**) 100 µM clioquinol or 300 µM HCQ with an additional 10 µM zinc chloride added to the media. Magnification ×10.

**Figure 13:**
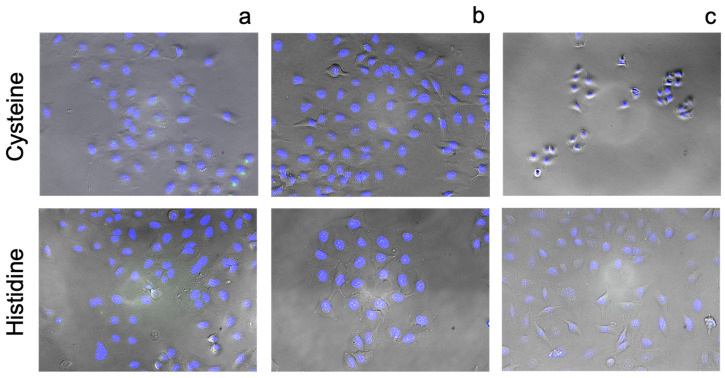
Fluorescence images of A549 cells incubated stained with FluoZin-3 AM (green) and counterstained using NucBlue Live for nuclei (blue) after 24 h incubation with: (**a**) 2.5 µM histidine or cysteine and basal zinc, (**b**) 2.5 µM histidine or cysteine and with an additional 10 µM zinc added to the media and (**c**) 300 µM histidine or cysteine with an additional 10 µM zinc added to the media. Magnification ×10.

**Figure 14:**
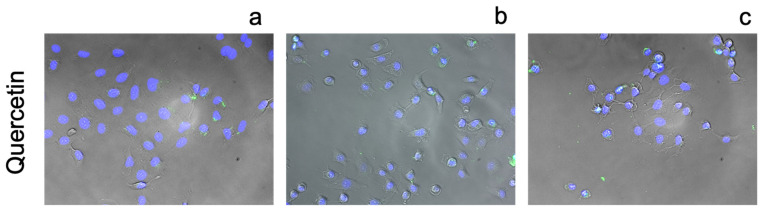
Fluorescence images of A549 cells incubated stained with FluoZin-3 AM (green) and counterstained using NucBlue Live for nuclei (blue) after 24 h incubation with: (**a**) 2.5 µM quercetin and basal zinc, (**b**) 2.5 µM quercetin with an additional 10 µM zinc chloride added to the media and (**c**) 100 µM quercetin with an additional 10 µM zinc chloride added to the media. Magnification ×10.

Figure 10, Figure 11, Figure 12, Figure 13 and Figure 14 illustrate results from cell transport studies. Initially we investigated the ability of each compound to increase the intracellular concentration of free zinc in basal media and subsequent experiments exposed the cells to excess levels of zinc and drug. These two conditions allow us to mimic two drug delivery routes, systemic (relatively lower concentrations) and topical (high excess concentrations).

Figure 10 illustrates that excess zinc does not disrupt the barrier function of the membrane and cell morphology appears unaffected. These results serve as a reference point for the other experiments.

Pyrithione (Figure 11) elicits a strong response from FluoZin-3 even at relatively low concentrations (0.125 µM). The addition of zinc to a solution containing pyrithione potentiates its toxicity, with a clear reduction in cell number. The rounded morphology of the remaining cells, in addition to profuse FluoZin staining, strongly suggests cell death. Increasing the concentration of pyrithione to 15 µM in the presence of additional zinc results in similar levels of toxicity.

With quinolones HCQ and CQL, 2.5 µM concentrations produce a strong fluorescent response from FluoZin-3 (Figure 12). Closer inspection of the images reveals that CQL has a more diffuse localisation of zinc whereas HCQ appears to localise zinc inside vesicular compartments, as described previously [13,58]. Interestingly, additional zinc and HCQ to concentration extremes does not cause quantitative changes to cell toxicities, unlike ‘true’ ionophores pyrithione and CQL.

Figure 13 illustrates that amino acids do not elicit increases of intracellular zinc in any of the conditions tested. This may reflect the utility of amino acids where absorption into the cell will result in their incorporation into proteins and peptides essential for cell growth; the fate of amino acids once entering cells has been described more completely in a number of reviews [59,60]. High concentrations of cysteine and histidine did induce cell toxicity through mechanisms which have been explored previously [61,62,63,64].

In agreement with previous results in a HeLa cell line [21], quercetin illustrates weak ionophoric activity (Figure 14). At higher concentrations, toxicity is apparent; quercetin and other polyphenols have demonstrated these effects on other mammalian cell lines [65,66].

## 4. Discussion

### 4.1. Identification of Effective Zinc Chelators with CSD Analysis

Interestingly, zinc–ligand distances in the CSD and those found in biology are strikingly similar [67]. Ireland and Martin also highlighted that cysteine and histidine residues are predominantly present in the coordination sphere of zinc in biological peptides and proteins. Although there is a clear utilisation of sulphur in biology, the same frequency is not found in the CSD; this may be explained by the fact that sulphur-containing compounds can be unpleasant to work with. Each compound group has been probed using the CSD to identify structural property relationships and binding modes in the solid state. This information could inform the development of more potent complexing agents which may possess ionophoric behavior in relation to zinc.

### 4.2. Quinolines

For medicinal chemists, quinoline is a common functionality that gained significant attention as a treatment for malaria [68]. In this disease state, the property of this group is to bind to hematin, inhibiting the formation of toxic 𝛽-hematin crystals deposited by the parasite [69]. The ability to chelate iron is conferred by the quinoline group, which also facilitates its localisation within the acidic lysosomal compartment [13,70,71,72,73]. These characteristics, along with promising experimental data from more modern quinolines, such as clioquinol [14] and PBT2 [15,16] (and its derivatives), which increase intracellular zinc levels, have led to much speculation about the zinc-binding activity of classic quinoline agents, such as chloroquine and hydroxychloroquine [17].

**Figure 15:**
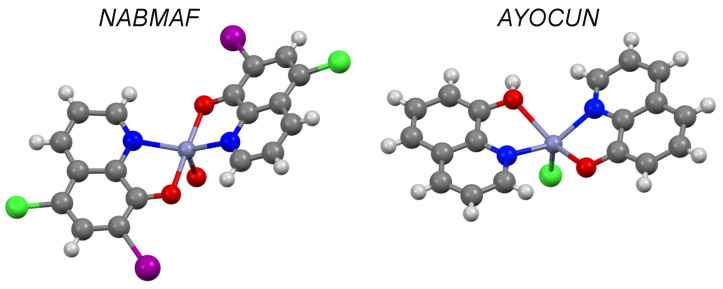
Solid state complex of clioquinol (NABMAF) and 8-hydroxyquinoline (AYOCUN) with zinc (purple). Other elements are coloured as follows: carbon (grey), oxygen (red), nitrogen (blue), hydrogen (off-white), chlorine (green) and iodine (purple).

Clioquinol (CQL) and 8-hydroxyquinoline form complexes with zinc in a 2:1 ratio (CSD refcodes: NABMAF and AYOCUN, Figure 15) where zinc is chelated between two inverted hydroxyquinoline molecules. 

Although zinc hydroxychloroquine complexes cannot be identified in the CSD, complexes between zinc and structural analogues (CSD refcode: AFIGAA) highlight the importance of the quinoline group for complexation with zinc. Further, chloroquine zinc complexes have been described in the literature where zinc is bound to the pyridine moiety [74] or to the secondary nitrogen [75]. Solid-state analysis highlights that the ancillary functionality may be important for the activity of complexation. As such, 8-hydroxychloroquine (8-HQ) and CQL might be expected to bind to zinc whereas hydroxychloroquine (HCQ)—without nearby donating groups on the quinoline ring—is less likely. Solid state analysis is supported by the literature reporting that complexation to zinc has been observed in 8-HQ and CQL [37] and has not been observed in HCQ. This suggests that, in general, quinoline rings chelate most effectively when they possess these additional donating groups and explains the lack of ionophorism in liposomal systems with HCQ. This also suggests that the detection (with FluoZin-3) of increased intracellular zinc caused by HCQ may not be through ionophoric mechanisms, but this will need to be explored further.

### 4.3. Flavonoids

**Figure 16:**
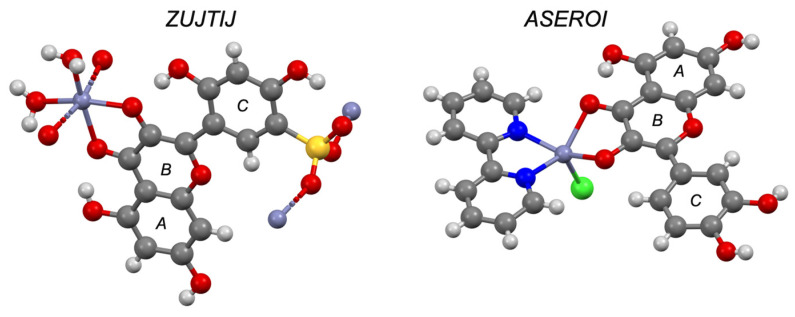
Solid-state complex of morin-5′-sulfonic acid (ZUJTIJ) and quercetin (ASEROI) with zinc (purple). Other elements are coloured as follows: carbon (grey), oxygen (red), nitrogen (blue), hydrogen (off-white), chlorine (green) and sulphur (yellow).

Flavonoids are found ubiquitously in plants where they are thought to play an important role primarily as antioxidants [76]. The metal complexes of their glycoside derivatives produce the wide colour palette seen in flowering plants [77,78]. Their presence in a variety of foods has been linked with a wide variety of nutritional and therapeutic benefits and they have been the subject of numerous reviews [76,79,80]. There are just 18 flavonoid–metal complexes on the CSD and in every complex zinc is bound between the carbonyl and hydroxyl groups on the B ring, even in the presence of competing catechol functionalities (CSD refcodes: ASEROI, ZUJTIJ and ZUJGUI, Figure 16).

Zinc binds to a variety of polyphenols in solution, and, once bound, zinc does not bind again to a different site on the same molecule [81]. The zinc complexation affinity of quercetin over other polyphenols has also been explored in an in depth NMR study by Primikyri et al. using another polyphenol analogue (lucetin); the authors further state that, in addition to the 1:1 complexation expected [82], they also found that zinc binds preferentially between the carbonyl and hydroxyl group on the B ring of quercetin. This has been described previously for a structurally similar flavonoid, myrecetin [81]. These results may provide an explanation for the contrasting activity between quercetin and naringenin, whereby the lack of a hydroxyl moiety on naringenin decreases its complexation strength with zinc and ultimately its ability to transport zinc across membranes. 

### 4.4. Polyols

**Figure 17:**
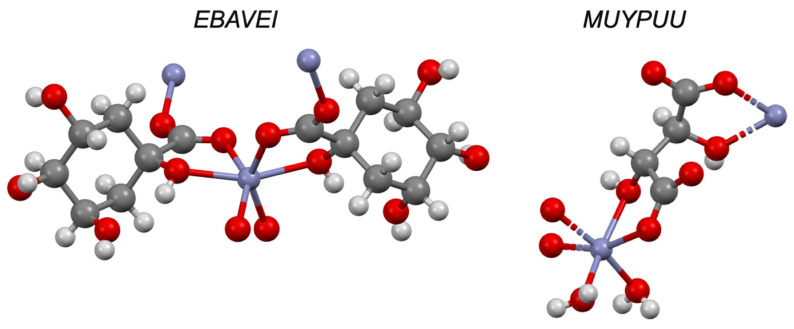
Solid-state complex of 1,3,4,5-tetrahydroxycyclohexanecarboxylic acid (EBAVEI) and tartaric acid (MUYPUU) with zinc (purple). Other elements are coloured as follows: carbon (grey), oxygen (red) and hydrogen (off-white).

Sugars are perhaps the most well-known polyols and their ability to complex with metals has been known for many years [83]; indeed, this effect has been exploited to increase the bioavailability of metals from the diet [84]. Carbohydrates have a long history in this space and their complexation with a variety of metals in solution (including zinc) has been reviewed extensively [85,86]. Work by Lim and colleagues has sought to synergise this increased bioavailability with the antimicrobial activity of specific polyols, such as erythriol [87]. In the CSD, a search of common sugars (mannitol, sorbitol, xylitol, erythriol, glycerol and glucose) returned just two structures of sugars with zinc: erythriol and glycerol (CSD refcodes: DADMEB and QQQAZD01, respectively). In these structures, one zinc molecule is bound by the hydroxyl groups of two sugar molecules, each contributing two hydroxyl sites to form a 2:1 complex. A wider search revealed that in molecules such as tartaric acid zinc will bind to one hydroxyl and one carboxylate moiety on each molecule, even in the presence of appropriately situated hydroxyl groups (CSD refcodes: EBAVEI, AQOHEU, FADJAX, ETUTUJ, MUYPUU and YEYKIX; Figure 17).

In erythromycin acetate (CSD refcode BOPRON10) zinc binds to the desosamine ring in spite of the presence of many hydroxyl groups on the lactone ring. Erythromycin zinc acetate (Zyneryt^®^) is a pharmaceutical product for the treatment of acne, and one author noted that “*The undoubted efficacy of topically applied Zineryt^®^ lotion in the treatment of acne cannot at present be explained by a single hypothesis. However, it is significant that this erythromycin-zinc complex promotes the penetration of zinc into the pilosebaceous units*” [88].

**Figure 18:**
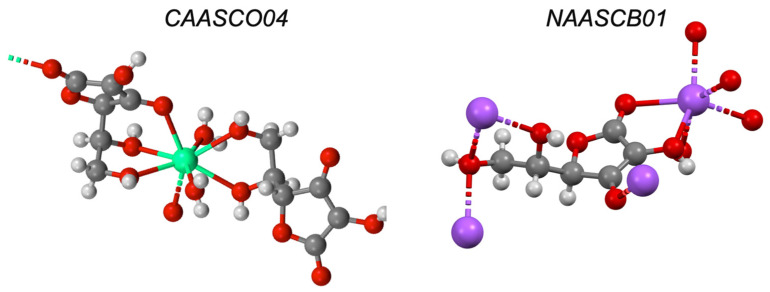
Solid-state complex of ascorbic acid and calcium (CAASCO04) and sodium (NAASCB01). Other elements are coloured as follows: carbon (grey), oxygen (red), hydrogen (off-white), calcium (aquamarine) and sodium (purple).

Ascorbic acid is known to form chelates with iron to improve its absorption [89,90]. This work has been extended to include other ions, such as zinc [39,91], where it is commonly seen as an adjuvant in zinc products for colds and flus. Although no crystal structure is known, combined experimental and computational analysis suggests a 2:1 complex stabilised by two hydroxyl groups on the lactone rings of one ascorbic acid and with the lactone carbonyl and one free hydroxyl group on another ascorbic acid species in the presence of two water molecules (i.e., a hexacoordinate) to form a 2:1 complex [39,91]. In the CSD there are several ascorbate salts with metals (calcium, CAASCO; sodium, NAASCB; lithium, PAJNOD; and thallium, TLASCB; Figure 18). Due to the conformational flexibility of polyols and the wide variety of metal–ion complexes, it is difficult to identify key structural features that could be modulated to improve ionophoric behaviour. However, their high water solubility (and the high water solubility of their corresponding metal complexes [89,90]) may reduce their capacity to transport ions across the non-polar membrane environment.

### 4.5. Amino Acids

**Figure 19:**
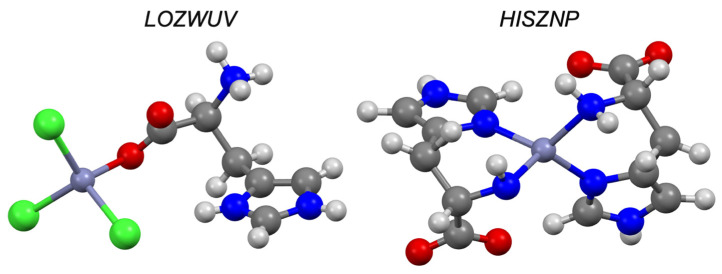
Zinc–histidine crystalline polymorphs obtained from the CSD. Other elements are coloured as follows: carbon (grey), oxygen (red), nitrogen (blue), hydrogen (off-white) and chlorine (green).

The wide variety of chemical diversity illustrated by amino acids make them the perfect candidates to investigate structural property relationships. They are particularly interesting candidates in this context as their function in biological systems can involve metal complexation, which is an essential prerequisite to enable the function of many key enzymes and proteins, e.g., matrix metalloproteases or zinc-fingers. CSD analysis revealed a total of 131 zinc amino acid complexes; almost all (n = 129; 98%) involved the carboxylate group. The two exceptions were zinc complexes with cysteine and histidine (CSD refcodes CURLUW and HISZNP01, respectively). Previous analysis of zinc binding sites in biological systems [67] has identified cysteine and histidine as the predominant amino acid residues involved in the majority of biological zinc complexes. The CSD analysis also revealed two distinct binding motifs (i) where zinc binds to a single carboxyl group on each amino acid in a chain-like fashion (n = 90; 69%) or (ii) binds with one carbonyl and the adjacent amino group on the same amino acid (n = 40; 31%); some structures have both motifs (CSD refcodes: GALSER, INOYAL, IVOVEU, JEMWIG and ZNGLUD01), and Figure 19 illustrates this in zinc–histidine polymorphs. All of the amino acids selected are capable of forming zinc complexes in the solution and solid states and some are widely available as commercial products, e.g., PolapreZinc^®^ (zinc–carnosine) and OptiZinc^®^ (zinc–methionine). These results confirm the importance of imidazole groups and terminal sulphur groups for the complexation of zinc and that the CSD can reveal alternate binding modes, particularly where competing functional groups are present on the same molecule. The affinity of histidine and cysteine for metal ions such as zinc comes as no surprise considering their biological function in zinc-finger proteins and metallothioneins. 

### 4.6. Imidazoles

In pharmaceuticals, the imidazole functionality is best known for its presence in the azole class of antifungal agents derived from triarimol [92]. Their mechanism is based on the inhibition of catalytic enzymes which enable the biosynthesis of ergosterol (essential to the integrity of the fungal cell wall) [93]. The mechanism of inhibition is through complexation with the catalytic haem group contained within the enzyme [94], which may prevent oxygen and peroxide activation by haem enzymes [94]. In biology, the imidazole functionality is essential to enabling the binding of metal ions in the catalytic sites of metalloproteases and structural proteins [95].

**Figure 20:**
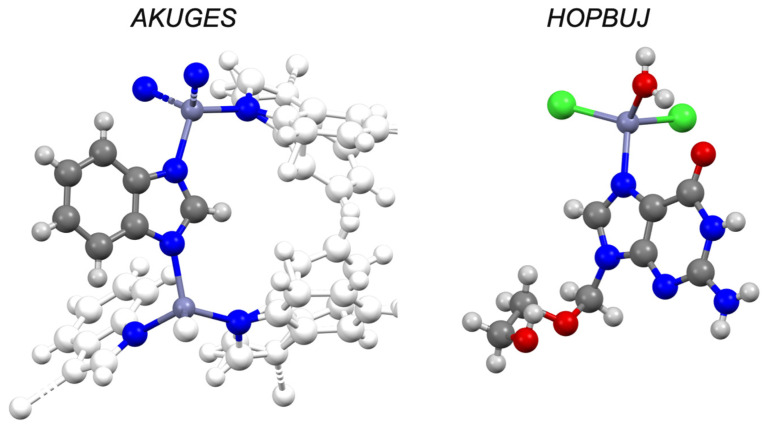
Solid-state complex of benzimidazole (AKUGES) and aciclovir (HOPBUJ) with zinc. Other elements are coloured as follows: carbon (grey), oxygen (red), nitrogen (blue), hydrogen (off-white) and chlorine (green).

Structural analysis (CSD) reveals a total of 1515 zinc complexes with imidazoles. Benzimidazoles account for a small fraction of this total (n = 282; 19%) and structural analogues, such as triazoles, have a total of 496 complexes with zinc. Aciclovir is capable of forming solid-state complexes with zinc through its imidazole functionality, and structural derivatives (i.e., guanosine; CSD refcodes: DAZTIH and WEWKEO) illustrate zinc binding at the same position. There are five structures in the CSD which have caffeine chelating to zinc (CSD refcodes: RITKUD, RITLEO, PUMRUL, LOYHAL and KASNUO), again demonstrating the affinity of zinc to the imidazole nitrogen (Figure 20). A zinc complex with levamisole is known, and although there are no X-ray structures with zinc deposited to the CSD, there are structures with platinum and nickel (CSD refcodes: LALGAG and JAXNUS, respectively), where the nitrogen of the fused imidazole ring is involved in complexation. Previous structural studies of the zinc levamisole complex suggested a similar binding mode [48]. Benzimidazole and its derivatives are known to complex with a wide variety of metals [96,97,98], with many structures deposited to the CSD, and in each case the imidazole nitrogen is responsible for binding. Our results confirm the utility of the imidazole group (a common functionality in pharmaceuticals) to chelate to zinc. The CSD reveals that zinc can bind to either nitrogen on the imidazole ring and that this chelation motif persists throughout a variety of functionalized imidazole compounds. This may explain why almost all of the imidazoles tested had some degree of ionophoric behaviour (although weak). 

### 4.7. Pyrithione

**Figure 21:**
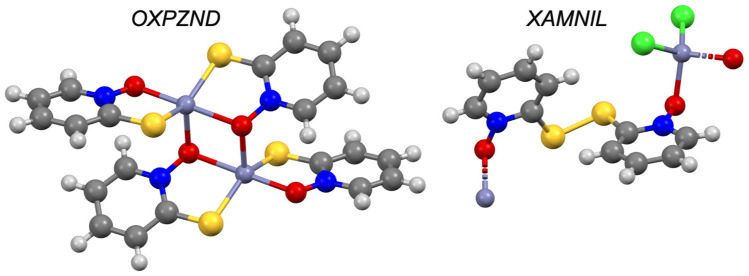
Solid-state complex of pyrithione (OXPZND) and 2,2′-dithiobis(pyridine N-oxide) (XAMNIL) with zinc. Other elements are coloured as follows: carbon (grey), oxygen (red), nitrogen (blue), hydrogen (off-white), chlorine (green) and sulphur (yellow).

Thirteen solid state complexes with zinc and pyrithione (and its analogues) have been deposited to the CSD where zinc binds between the oxygen of the amine oxide group and the thiol of two pyrithione molecules in a trans geometry. Three exceptions are noted in the same publication (CSD refcodes: XAMNIL, XAMNOR and XAMNUX; Figure 21), in which the authors employ the use of a disulfide bridge to disrupt the formation of the pyrithione dimer which chelates zinc [99]. In aqueous solution, evidence suggests that pyrithione forms a 1:1 complex [100]. Pyrithione is perhaps the oldest and most well-established medicinal zinc ionophore. While the exact mechanism is still being understood [33,34,35,36], pyrithione is known as an extremely potent agent [101], particularly when formulated as its zinc salt [36]. Pyrithione is a well characterized ionophoric agent and ultimately serves as a positive control for the experimental procedures. The CSD suggests that whenever sulphur is free, zinc will bind between it and the amine oxide group.

In this work, we identify terminal sulphur, imidazole, polyphenol and hydroxyquinolines as important functional groups which form complexes with zinc in the solid state and in solution. Many of these groups are found in species of biological importance (e.g., zinc-finger proteins and metallothioneins). We also find similarities between biological and synthetic zinc complexes and a lack of synthetic compounds utilizing sulphur functionalities to complex zinc. Our CSD analysis alongside the positive liposomal assays suggests that sulphur-containing functionalities may be relatively underexplored within the context of zinc complexation and ionophorism. This activity could be augmented with additional donating groups, such as those seen in pyrithione or in the quinolines. It is important to note, however, that complexation in the solid state does not necessarily translate to solution and, further, complexation in the latter states will not always confer ionophoric properties.

Interestingly, we find that although HCQ can increase intracellular zinc concentrations, the exact mechanism is unclear, as there is no activity in liposomal assays. This behaviour was also seen with erythromycin [88] and may be due to a lack of sufficient counterions in solution or to the fact that transport is limited by cell potential. Another hypothesis is that erythromycin and HCQ alter intracellular zinc dynamics through interactions at protein targets (e.g., storage peptides, such as zinc-fingers). On the other hand, amino acids (particularly histidine and cysteine) demonstrate strong ionophorism in liposomes that is not translated to cellular assays. We plan to investigate this anomalous behaviour in further studies. More complex assays, particularly for the weaker ionophores in the imidazole class, may be necessary to highlight the clinical value of potential drug–zinc formulations. This is due to the difficulties associated with replicating the environment in vitro without causing significant toxicity prior to determining intracellular zinc concentrations. Examples include aciclovir and zinc formulations in the treatment of herpes simplex and Zyneryt^®^ (erythromycin zinc acetate) for acne.

## 5. Conclusions

Ultimately, we highlight the importance of *in cellulo* assays to confirm ionophoric activity. Although liposomal assays are important as a screening tool, they have limitations, e.g., they may underestimate ionophorism where another counter-ion is important to the ionophoric mechanism or overestimate ionophorism where efflux pumps and other biological species can neutralize the accumulation of ions intracellularly. Cellular systems have greater complexity and allow researchers to identify these false positives and false negatives. Future research into ionophoric compounds could explore biomimetic 3D cages which may have the added advantages of increased selectivity and specificity for zinc. Indeed, it has been identified that ionophores that form 3D cages are more effective compared to smaller, planar molecules [8].

It is still unclear what the implications of increased intracellular zinc are for physiology or pathophysiology. Further, the lack of specificity and sensitivity of small molecule ionophores may result in the transport of other ions and this could make it more difficult to elucidate precise transport mechanisms, as evidenced with the much-explored zinc ionophore pyrithione [33,34,35,36].

## Figures and Tables

**Table 1:** Summary of physicochemical and experimental data grouped by chemical functionality.

Drug	Log P_7.4_^a^	BCS	Solid-State Complex ^b^	Solution Complex	Liposomal Ionophorism	
					0.1 mM	1 mM
**Quinolines**						
8-hydroxyquinoline ^d^	1.82	I	AYOCUN	✓ [37]	-	✓
Clioquinol ^d^	3.03	II	NABMAF	✓	✓	
Hydroxychloroquine ^d^	0.33	I	-	-	-	-
**Polyols**						
Ascorbic acid ^e^	−5.00	I	✓ [38]	✓ [39]	✓	
Erythromycin ^d^	0.99	III	BOPRON10	✓ [40]	-	-
Zinc gluconate ^e^	-	I	✓ [41]	✓ [41]	-	✓
**Flavonoids**						
Naringenin ^e^	2.70	II	✓ [42]	✓ [43]	-	✓
Quercetin ^e^	1	II/IV	ASEROI	✓ [44]	✓	
**Imidazoles**						
Aciclovir ^d^	−1.03	IV	HOPBUJ	✓ [45]	-	✓
Mebendazole ^d^	3.25	II	-	✓ ^c^ [46]	-	✓
Caffeine ^d^	−0.55	I	RITLEO	✓ [47]	-	✓
Levamisole ^d^	2.22	I	✓ [48]	✓ [48]	-	-
**Amino acids**						
Carnosine ^d^	−4.51	I	✓ [49]	✓ [50]	-	-
Cysteine ^e^	−2.80	I	CURLUW	✓ [51]	✓	
Histidine ^e^	−3.64	I	MUYFEU	✓ [51]	✓	
Methionine ^e^	−2.19	I	LMETZN01	✓ [52]	-	-
Proline ^e^	−2.57	I	HIBTOB01	✓ [53]	-	-
Tryptophan ^e^	−1.09	I	✓ [54]	✓ [55]	-	✓
**Miscellaneous**						
Pyrithione ^d^	−0.41	III	OXPZND [56,57]	✓ [8]	✓	

Biopharmaceutics classification system (BCS). ✓ = positive result. ^a^ LogP at pH 7.4 calculated using ChemAxon. ^b^ CSD refcode provided where available. ^c^ Mebendazole derivatives. ^d^ Pharmaceutical. ^e^ Nutraceutical. A hyphen denotes when an experiment was performed but failed to demonstrate activity.

## Data Availability

Data available on request.
